# Preparation of Antarctic Krill Oil Emulsion and Its Stability under Catalase Treatment

**DOI:** 10.3390/foods10112797

**Published:** 2021-11-13

**Authors:** Zhenxiao Zheng, Kai Zhu, Zhiyuan Dai

**Affiliations:** 1 Institute of Seafood, Zhejiang Gongshang University, Hangzhou 310012, China; zzx@zjgsu.edu.cn (Z.Z.); kkkaizhu@163.com (K.Z.); 2 State Key Laboratory of Aquatic Products Processing of Zhejiang Province, Hangzhou 310012, China; 3 Collaborative Innovation Center of Seafood Deep Processing, Institute of Seafood, Zhejiang Gongshang University, Hangzhou 310012, China

**Keywords:** Antarctic krill emulsion, catalase, iron porphyrin, lipid oxidation

## Abstract

Making Antarctic krill oil into emulsion is a good way to utilize Antarctic krill, but proliferation of microorganisms cannot be ignored. H_2_O_2_ is widely used in the sterilization of liquid food since its decomposition products are environmentally friendly, although residual H_2_O_2_ should be removed for food safety. Adding catalase (CAT) is an effective means to do this. However, the enzyme activity center of CAT is the iron porphyrin group, which has the risk of accelerating lipid oxidation in the oil emulsion. Therefore, we hypothesized that CAT might not be suitable for the removal of H_2_O_2_ in Antarctic krill oil emulsion. In this paper, Antarctic krill oil emulsion was prepared, and then the effect of CAT on the emulsion was studied through visual observation, microscopic morphology observation, turbidity and stability, particle size, and ζ-potential; finally, the mechanism of CAT destroying the emulsion was explored from the perspective of lipid oxidation. The results showed that a stable Antarctic krill emulsion was prepared using Tween-80 as the emulsifier, with the oil concentration of 1% (*v*/*v*) and the ratio of surfactant to oil phase of 1:5 (*v/v*). The emulsion treated with CAT had undergone demulsification, stratification, and coagulation after 2 days of incubation, while the emulsion treated with superoxide dismutase (SOD) and bovine serum albumin (BSA) changed little. In addition, the thiobarbituric acid reactive substances (TBARS) value and the content of hydroxyl radicals in the CAT group increased significantly. The preliminary research results indicated that the effect of CAT on the emulsion related to the lipid oxidation caused by the iron porphyrin group at the center of the enzyme activity. All these results indicated that CAT was not suitable for the removal of residual H_2_O_2_ in Antarctic krill oil emulsion. Moreover, it is helpful to avoid the contact of Antarctic krill oil emulsion and CAT during the processing of the krill.

## 1. Introduction

Antarctic krill (*Euphausia superba*) is a type of crustacean plankton that lives in the Antarctic Ocean and has attracted strong research interest from all over the world. It is reported that the resource storage of Antarctic krill is about 800 million tons [1], with an annual post-larval production of 342 to 536 million t. The annual catch of Antarctic krill is about 250,000 t, which is only about 1/3 of its catch limit set by the Commission for the Conservation of Antarctic Marine Living Resources (CCAMLR). Therefore, there is great potential for the development of global Antarctic krill resources [2]. Antarctic krill is usually utilized by being processed into bait used in aquaculture industries [3]. However, due to the limited scale of aquaculture and the variety of bait resources, expanding the utilization ways of Antarctic krill resources is becoming increasingly important. In recent years, with the increasing recognition of the nutritional value of Antarctic krill, processing Antarctic krill into functional food has become an important method of high-value utilization. Antarctic krill oil is rich in phospholipid-bound *n*-3polyunsaturated fatty acid (*n*-3PUFA). Compared with the glyceride *n*-3PUFA in fish oil, phospholipid-bound *n*-3PUFA fuses better with cell membranes and is absorbed more easily [4,5]. Therefore, Antarctic krill is a good resource for preparing functional oils.

However, due to the higher content of unsaturated fatty acids in Antarctic krill oil, oxidative rancidity is prone to occur, which greatly affects the quality and commercial value of the oil [6]. Moreover, the high viscosity and the strong unpleasant smell of Antarctic krill oil also increase the difficulty in the processing of Antarctic krill oil. Making Antarctic krill oil into an emulsion is a promising way to solve these problems. Yet, the proliferation of microorganisms during storage will also cause the deterioration of the emulsion quality [7]. H_2_O_2_ is widely used in the sterilization of liquid food as its decomposition products are environmentally friendly and residual H_2_O_2_ should be removed for food safety [8,9]. Adding catalase (CAT) is an effective means to do this. Therefore, research on the effect of CAT on the stability of Antarctic krill oil emulsion should be carried out prior to CAT application. For this, we searched relevant literature. The current research on the factors affecting the stability of oil emulsions mainly focused on preparation methods and types of emulsifiers. Akbas et al. [10] prepared a nanoemulsion by high pressure homogenization and ultrasonic crushing. It was found that emulsion prepared by high-pressure homogenization had higher embedding capacity than that prepared by ultrasonic crushing. Jiang et al. [11] prepared fish oil a nanoemulsion using soybean-protein–phosphatidylcholine as a composite emulsifier. The emulsion was stable when stored at 4 °C and 25 °C for 30 days and had better storage stability than Tween-20 fish oil nanoemulsion. Studies on the effect of CAT on the stability of Antarctic krill oil emulsions are rare. For CAT the active center of this enzyme is iron-containing porphyrin. When CAT functions, the iron element continuously switches between low and high valence to complete the electron transfer, and the iron element is in a very active state, increasing the risk of electron omissions from the porphyrin [12]. Free electrons are fatal to grease. They can trigger the oxidative rancidity of grease even at a very low concentrations [13]. Based on this, we hypothesized that CAT might not be suitable for the removal of residual H_2_O_2_ in Antarctic krill oil emulsion.

When preparing the emulsion, it is very important to choose suitable surfactants. Appropriate emulsifiers can reduce the interfacial tension and are conducive to the formation of small emulsion droplets [14]. Emulsions with smaller droplets are more stable than larger ones [15,16]. Tween-80 is a non-ionic surfactant, usually used in the preparation of oil-in-water emulsions, while Span-20 is a non-ionic surfactant used in water-in-oil emulsion preparation. Lecithin is an ionic surfactant which is widely used in the food industry. In this article, these three surfactants were used to prepare emulsions and the appropriate surfactant was selected based on the characterization index of the emulsion. In addition, the ratio of surfactant to oil concentration is also crucial to the stability of emulsion. Both a too-high and a too-low ratio of surfactant to oil will destroy the dynamic equilibrium of emulsion [17]. Oil concentration is also crucial. In this paper, the effects of different oil concentration (0.25%, 0.50%, 1.00%, 2.50%, 5.00%, *v*/*v*) and the ratio of surfactant to oil (1:10, 1:5, 1:1, 5:1, 10:1, *v*/*v*) on the state of the emulsion were evaluated and the appropriate values were selected. Furthermore, the effects of different concentrations of CAT on the stability of Antarctic krill oil emulsion during storage were discussed through visual observation, microscopic morphology observation, turbidity and stability, particle size and ζ-potential. Finally, the mechanism by which CAT affects the stability of Antarctic krill emulsion was primarily explored from the perspective of lipid oxidation. We hope this work will be helpful in the rational utilization of Antarctic krill resources and provide information about processing of Antarctic krill oil emulsion.

## 2. Materials and Methods

### 2.1. Material, Chemicals and Reagents

The Antarctic krill oil was provided by Liaoyu Group Co., Ltd., Dalian, China, China. The composition of the Antarctic krill oil is listed in Tables S1 and S2. Tween-80, lecithin and Span-20 were purchased from Sinopharm Co., Ltd., China. Bovine liver catalase (CAT, C128522), superoxide dismutase from bovine erythrocytes (SOD, S128537), and bovine serum albumin (BSA, A116563) were obtained from Aladdin Co., Ltd., China. 

### 2.2. Preparation of Antarctic Shrimp Oil Emulsion

A certain amount of surfactant was dissolved into ultrapure aqueous solution. Antarctic krill oil was slowly added to the system and then the mixture was homogenized at 18,000 rpm for 3 min. Emulsions were prepared with different emulsifiers (Tween-80, lecithin, and Span-20), different oil concentrations, and different ratios of surfactant to oil. The preparation conditions were optimized by the indices of turbidity, stability, particle size and ζ-potential. 

### 2.3. CAT Affected the Status of Antarctic Shrimp Oil Emulsion

The Antarctic shrimp oil emulsion was prepared at the optimal conditions. CAT SOD, and BSA solutions were added to the emulsion and the final concentrations were 0.25 mol/L, 0.50 mol/L, 1.00 mol/L, 2.50 mol/L, 5.00 mol/L. Then, they were incubated at 37 °C. The effect of CAT, SOD and BSA on the emulsion were evaluated by visual and micromorphological observation, turbidity, stability, particle size and ζ-potential.

### 2.4. CAT Activity and Emulsion Stability

Emulsions containing 0.50 μmol/L, 1.00 μmol/L, and 5.00 μmol/L of normal CAT and inactivated CAT were prepared. The emulsions were incubated at 37 °C and the turbidity was measured. The aqueous solution and Antarctic krill emulsion containing 0.50 μmol/L, 1.00 μmol/L, and 5.00 μmol/L of CAT were prepared, and incubated at 37 °C. Changes in enzyme activity during the incubation period were measured. 

### 2.5. CAT Affected the Lipid Oxidation of Antarctic Shrimp Oil Emulsion

Different concentrations (0, 0.50 mol/L, 1.00 mol/L, and 5.00 mol/L) of CAT and BSA were added to the emulsion and they were incubated at 37 °C. The impact of CAT on the lipid oxidation of Antarctic shrimp oil emulsion was evaluated by TBARS and hydroxyl radicals.

### 2.6. Measurement of the Turbidity and Stability

After the emulsion was diluted 10 times, the absorbance value of the emulsion was measured at 600 nm by UV/VIS spectrophotometer (Evolution350, ThermoFisher, Waltham, MA, USA) and turbidity Aa was obtained by parallel determination. This was repeated three times. The stability of emulsion was measured by the centrifugation method. The emulsion was centrifuged at 3000 rpm for 10 min and the supernatant was measured at 600 nm to obtain Ab. The stability of the emulsion was calculated by Equation (1).

Stability = Ab/Aa × 100%(1)

Note: “Ab” and “Aa” are refferd to the absorbance of the emulsion before and after the centrifugation.

### 2.7. Measurement of the Particle Size and ζ-Potential

We took a certain volume of the sample and placed it in the analysis room of ZetaSizer Nano-ZS instrument (Malvern Instruments Co. Ltd., Worcestershire, UK). We set the determination conditions and started the determination after 60 s of equilibrium. The particle size and ζ-potential were read from the instrument.

### 2.8. Transmission Electronic Microscopy (TEM) Observation

The emulsion was dripped onto 300-mesh copper mesh coated with Formvar film and the excess emulsion was removed from the edge of the copper mesh. Negative staining was carried out with phosphotungstic acid (1%). After absorbing the excess emulsion with filter paper, the morphology and size of the milk droplets were observed on a transmission electron microscope (JEM-1230, JELO, Tokyo, Japan) at an accelerating voltage of 80 kV.

### 2.9. Measurement of the CAT Activity

The CAT activity was measured through a kit (A007-1-1) provided by Nanjing Jiancheng Bioengineering Institute, Nanjing, China. The measurement process was conducted according to the instructions. 

### 2.10. Measurement of the TBARS

The detection of TBARS was carried out according to the method of McDonald and Hultin [18]. First, 1.0 mL of emulsion was mixed with 2.0 mL thiobarbituric acid (TBA) reagent and incubated in boiling water bath for 15 min. Then, the sample was placed in an ice-water bath and cooled rapidly to room temperature. After 1000 rpm centrifugation for 10 min, the absorption value of the supernatant was measured at 532 nm. The concentration of TBARS was determined according to the standard curve of 1,1,3,3-tetraethoxypropane.

### 2.11. Measurement of the Hydroxyl Radicals

The detection of hydroxyl radicals was carried out according to the method of Du et al. [19]. A certain amount of terephthalic acid (TPA) was added to the emulsion to trap the hydroxyl radicals. The mixtures were directly exposed to the air and incubated for 24 h on a shaking bed (170 rpm), and then filtered via 0.22 μm filter membranes. The production of hydroxyl terephthalic acid (HTPA) was detected by HPLC.

### 2.12. Statistical Analysis

Differences between treatments (means ± SD, *n* = 3) were assessed with one-way analysis of variance (ANOVA), using Tukey’s honest significance test (HSD). The significance level was set at *p <* 0.05. Statistical analyses were performed using SPSS software version 19.0 for Windows (SPSS, Chicago, IL, USA). 

## 3. Results 

### 3.1. Optimization of the Antarctic Shrimp Oil Emulsion Preparation Conditions

#### 3.1.1. Impacts of Different Emulsifiers on the Emulsion Properties

First, 100 μL of surfactant (Tween-80, lecithin and Span-20), 250 μL Antarctic krill oil, and a certain amount of ultrapure aqueous were mixed (the final volume was 50.00 mL). Then, the mixture was homogenized at 18,000 rpm for 3 min. The property indices of the emulsion were measured (Table S3). The absorbance of the emulsion prepared by Tween-80, lecithin, and Span-20 was 0.56, 0.45, and 0.64, respectively. The stability was 75.16%, 42.37%, and 69.19%, respectively. The particle size of the emulsion prepared by Tween-80, lecithin, and Span-20 was 145.1 nm, 378.6 nm and 184.2 nm, respectively. The emulsion prepared by Tween-80 had the smallest particle size, which indicted that Tween-80 could fully emulsify the oil droplets and inhibit the polymerization of emulsion droplets, thereby making the emulsion particle size smaller. The ζ-potential of emulsion prepared by Tween-80, lecithin, and Span-20 was −40.6 mV, −68.2 mV and −35.1 mV, respectively. The absolute ζ-potential of emulsion prepared by lecithin was the highest, followed by Tween-80, and Span-20. Although the absolute ζ-potential of the emulsion prepared with lecithin was much higher than that of Tween-80 or Span-20, lecithin is an ionic surfactant which can generate anions or cations through ionization. Therefore, the ζ-potential of the emulsion with lecithin cannot truthfully reflect the state of the emulsion. Overall, the emulsion prepared by Tween-80 had the highest stability and the smallest particle size; the turbidity and ζ-potential were also appropriate. Tween-80 was selected as the best emulsifier.

#### 3.1.2. Impacts of Different Oil Concentrations on Emulsion Properties

First, 100 μL of Tween-80, different volumes of Antarctic shrimp oil (0.25%, 0.50%, 1.00%, 2.50%, and 5.00%) and ultrapure aqueous were mixed (the final volume was 50.00 mL). Then the mixture was homogenized at 18,000 rpm for 3 min. The property indices of the emulsion were measured (Table S4). The stability of the emulsion first increased and then decreased with the increase of the oil concentration and the maximum value was obtained at 1.00%. The particle size of the emulsion presented the opposite tendency. The minimum value was reached at 1.00%. The absolute ζ-potential of the emulsion increased with the increase of the oil concentration. The minimum (10.2 mV) and maximum (30.2 mV) value was obtained at 0.25% and 5.00%. Taking all factors into account, 1.00% was selected.

#### 3.1.3. Impacts of Different Ratios of Surfactant to Oil on the Emulsion Properties

First, 500 μL of Antarctic shrimp oil and different volumes of Tween-80 and ultrapure aqueous were mixed (the final volume was 50.00 mL). Then the mixture was homogenized at 18,000 rpm for 3 min. The characteristic indices of the emulsions prepared with different ratios of Tween-80 to oil is listed in Table S5. When the ratio of Tween-80 to oil was 1:10, the concentration of Tween-80 was too low to completely cover the oil droplets. The excess oil droplets were dispersed in the water phase, increasing the probability of aggregation. Therefore, the emulsion had high turbidity and low stability. With the increase of the ratio of Tween-80 to oil, the turbidity of the emulsion decreased and the stability increased. When the ratio of Tween-80 to oil was 1:5, a stable emulsion was formed. When the ratio was over 1:1, the excess emulsifier was dispersed in the aqueous phase, the turbidity increased, and the stability decreased. The particle size of emulsion first decreased and then increased with the increase of the ratio. When the ratio was below 1:1, the particle size of emulsion was smaller. When the ratio was over 1:1, the increase of free emulsifier in the aqueous phase led to uneven particle-size distribution and particle-size increase. As for the ζ-potential, the absolute ζ-potential decreased with the increase of the ratio, indicating that surfactant reduced the interfacial tension of emulsion droplets. After comprehensive consideration, the ratio 1:5 was selected.

#### 3.1.4. Preparation of Emulsion at Optimal Conditions

First, 100 μL Tween-80, 500 μL Antarctic krill oil and ultrapure water was mixed (the final volume was 50.00 mL). Then, the mixture was homogenized at 18,000 rpm for 3 min. The property indices of the emulsion were measured. The turbidity and stability of the emulsion were 0.65 and 75.63%, respectively. The average particle size and ζ-potential were 129.6 nm and 27.2 mV, respectively. These data indicated that the emulsion was stable.

### 3.2. Impacts of CAT on the Emulsion Status

#### 3.2.1. Visual Observation

**Figure 1:**
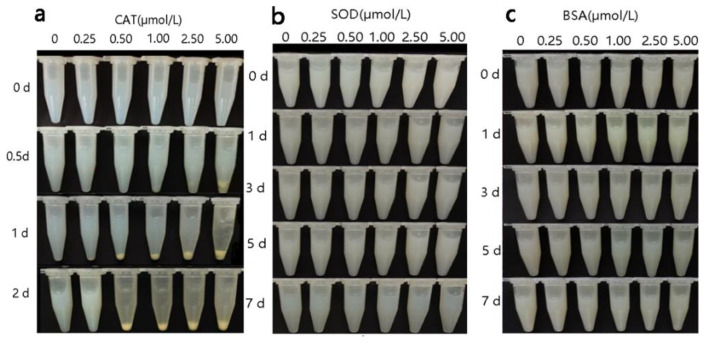
Visual observation changes of the Antarctic krill oil emulsion after catalase (CAT) (**a**), superoxide dismutase (SOD) (**b**), and bovine serum albumin (BSA) (**c**) treatment.

The appearance changes of emulsions treated with different concentrations of CAT, SOD, and BSA are shown in Figure 1. Lamination phenomenon was observed in the emulsion treated with a high concentration of CAT (5.00 μmol/L) after 0.5 day of incubation, and the stability of the emulsion gradually deteriorated. Moreover, after 2 days of incubation, stratification and precipitation were observed in the CAT treatment groups (except the 0.25 μmol/L group) and the emulsion was no longer homogeneous. For the SOD and BSA groups, the state of the emulsion was affected little. After 7 days of incubation, the emulsion still remained homogeneous. 

#### 3.2.2. Micromorphology Observation

**Figure 2:**
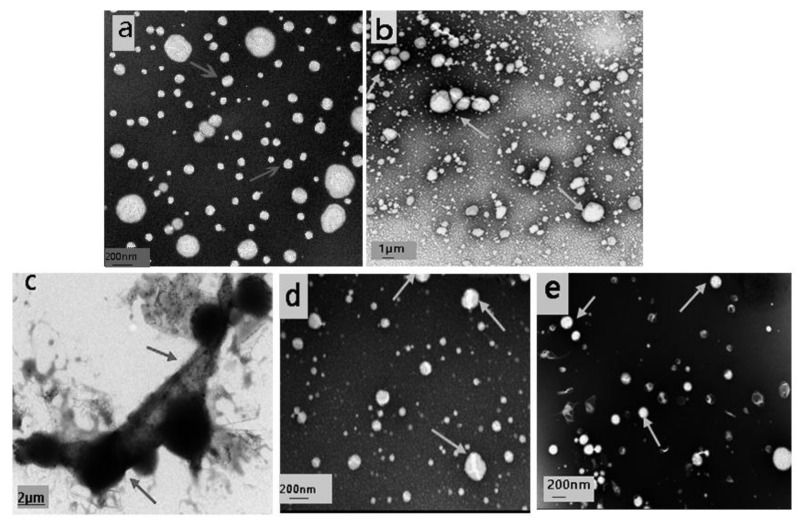
Microscopic morphology observation changes of the Antarctic krill oil emulsion after different treatments. (**a**) Emulsion incubated for 2 days, (**b**) supernatant of the emulsion treated with CAT (1.00 μmol/L) for 2 days, (**c**) precipitates of the emulsion treated with CAT (1.00 μmol/L) for 2 days, (**d**) the emulsion treated with SOD (1.00 μmol/L) for 2 days, and (**e**) the emulsion treated with BSA (1.00 μmol/L) for 2 days.

The TEM images of the emulsions with different treatment are shown in Figure 2. The particles of the emulsion itself were relatively uniform, in a regular spherical shape, and most of them were nano-level and evenly distributed in the emulsion system (Figure 2a). In the emulsion treated with CAT (1.00 μmol/L) for 2 days, particles in the emulsion tended to aggregate, the particles size became larger, and most of the particles were no longer nano-level (Figure 2b). This phenomenon was more obvious in the precipitation of the emulsion treated with CAT after 2 days incubation (Figure 2c). For the SOD (Figure 2d) and BSA (Figure 2e) groups, the particles in the emulsion were still relatively uniformly dispersed in the system, and no obvious coagulation was observed.

#### 3.2.3. Turbidity and Stability Changes 

**Figure 3:**
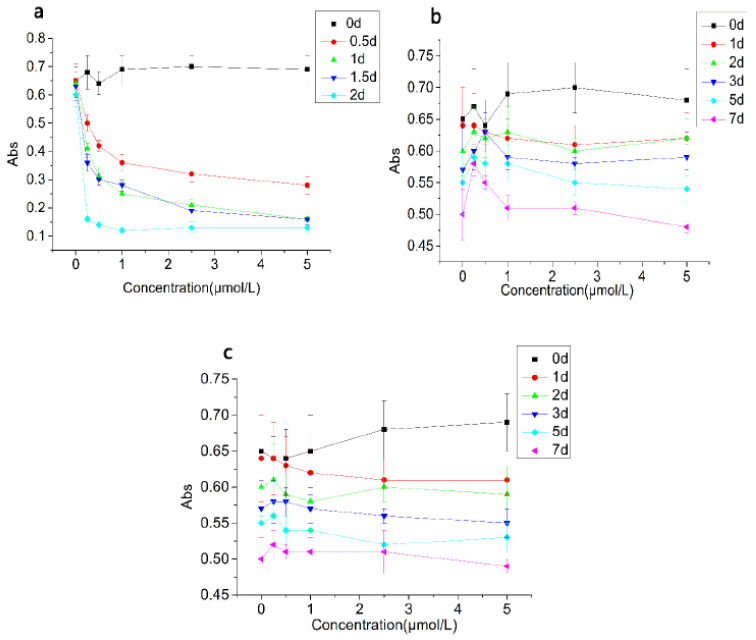
Turbidity changes of the emulsion treated with CAT (**a**), SOD (**b**), and BSA (**c**). Note: “Abs” is the absorbance of the emulsion at 600 nm.

**Figure 4:**
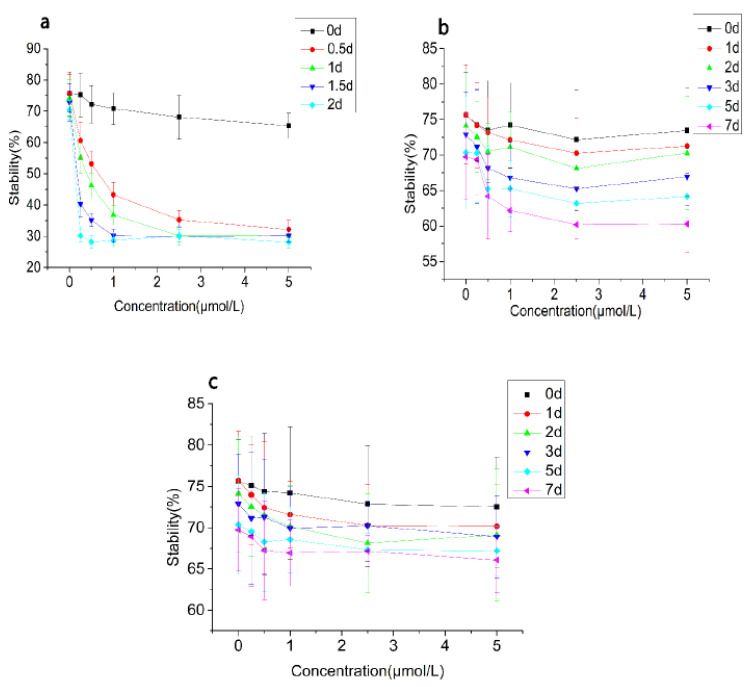
Stability changes of the emulsion treated with CAT (**a**), SOD (**b**), and BSA (**c**).

Turbidity and stability changes of the emulsion treated with CAT, SOD, and BSA are shown in Figure 3 and Figure 4. The turbidity of the emulsion gradually decreased after the CAT was added. The turbidity of emulsions treated with CAT was below 0.20 after 2 days of incubation, indicating that the emulsion system had been destroyed. In addition, when the concentration of CAT was below 1.00 μmol/L, the increase of the CAT concentration accelerated the decrease of the turbidity, while the decrease of turbidity correlated little with the amount of CAT added when the concentration of CAT exceeded 1.00 μmol/L. This result showed that the reaction between the emulsion and CAT tended to be saturated when the CAT concentration was 1.00 μmol/L. In terms of the stability, it decreased over time. The stability of the emulsion with high concentration of CAT decreased faster than that of the low concentration and the stability of groups decreased to about 30% on the second day.

The turbidity of the SOD treatment group also showed a gradual decrease over time, but the decreasing trend was far less than that of the CAT treatment group. The initial turbidity value of the SOD treatment group was 0.65–0.70. After 7 days of incubation, the turbidity value dropped to 0.48 to 0.58. The stability of the SOD treatment group was consistent with the results of the turbidity. The turbidity of the emulsion in the BSA treatment group changed little. The initial value of the turbidity was 0.65–0.69. After 7 days of incubation, the turbidity value was 0.49–0.52. The stability result was consistent with the turbidity.

In general, the effect of CAT on the emulsion was significantly stronger than that of SOD and BSA. After 2 days of incubation, lamination and coagulation were observed in the emulsions treated with CAT. For SOD and BSA groups, the emulsion changed little.

#### 3.2.4. Particle Size and ζ-Potential Changes 

**Figure 5:**
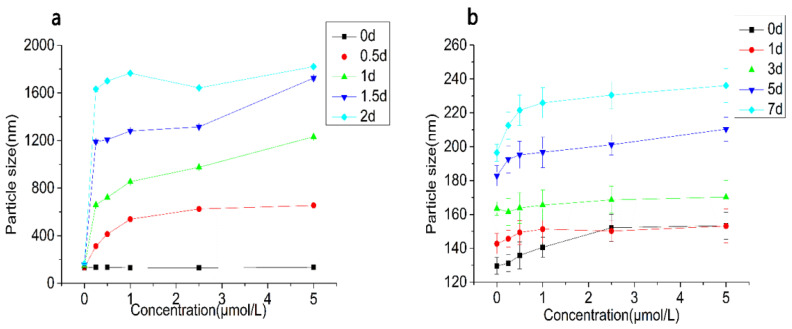

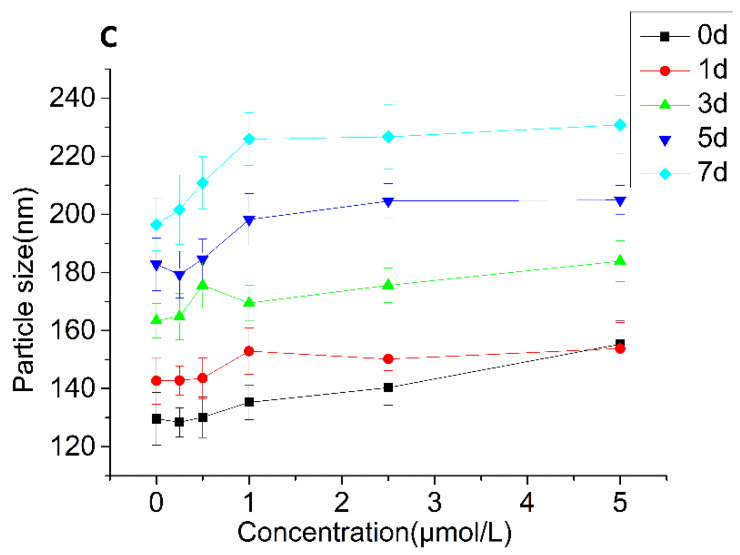
Particle size changes of the emulsion treated with CAT (**a**), SOD (**b**), and BSA (**c**).

**Figure 6:**
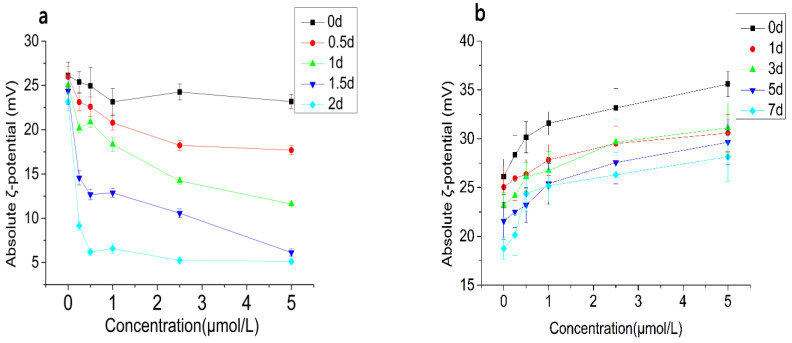

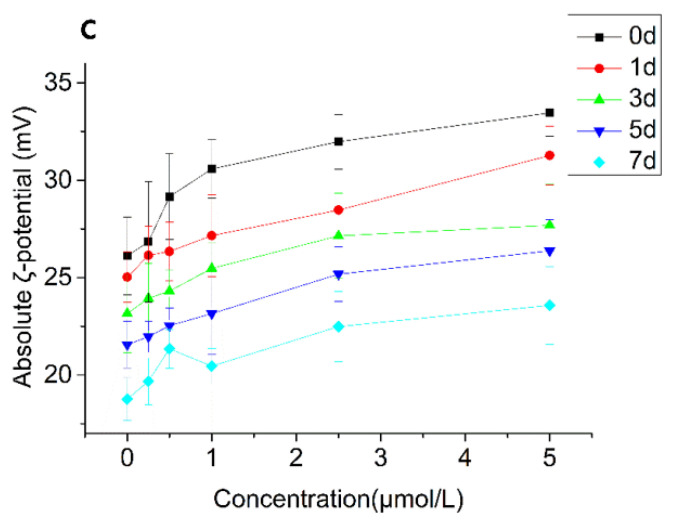
Absolute ζ-potential changes of the emulsion treated with CAT (**a**), SOD (**b**), and BSA (**c**).

In order to further characterize the emulsion, the particle size and ζ-potential of emulsions treated with CAT, SOD, and BSA were measured (Figure 5 and Figure 6). The particle size of emulsions treated with CAT increased rapidly with the incubation time, and the increasing rate accelerated with the increase of CAT concentration. After 1.5 days of incubation, the particle size of the emulsion exceeded 1000 nm and it exceeded 1500 nm after 2 days of incubation. After 2 days of incubation, the stability of the emulsion was destroyed, the original nano-level emulsion droplets were broken, and the emulsion droplets aggregated and settled, leading to the increase in the particle size. In terms of the absolute ζ-potential value, the value of the emulsion itself was about 27.20 mV. After adding CAT, the values of the emulsion decreased to varying degrees. The values of the emulsion even fell below 10 mV after 2 days of incubation. The particle size of the emulsions treated with SOD also increased to a certain extent with the extension of the incubation time, but it was not obvious. The particle size of the emulsion was still below 250 nm at the end of the experiment. The absolute ζ-potential of the SOD treatment group gradually decreased with the incubation time, but the decrease in speed was far less than that of the CAT treatment group. The changes in particle size and ζ-potential in the BSA treatment group were similar to those in the SOD treatment group.

### 3.3. Correlation Analysis between CAT Activity and Emulsion Stability and Lipid Oxidation

#### 3.3.1. CAT Activity and Emulsion Stability

**Figure 7:**
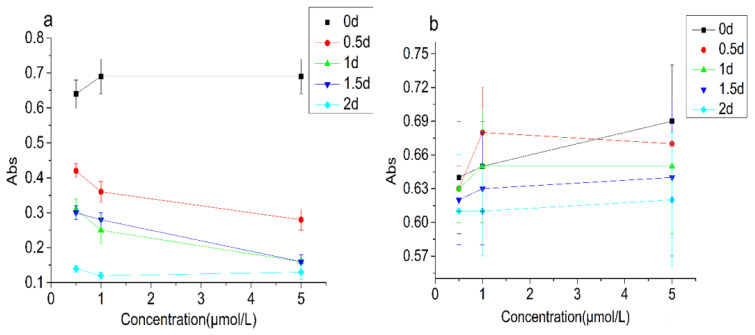
Turbidity changes of the emulsion treated with CAT (**a**) and inactivated CAT (**b**).

**Figure 8:**
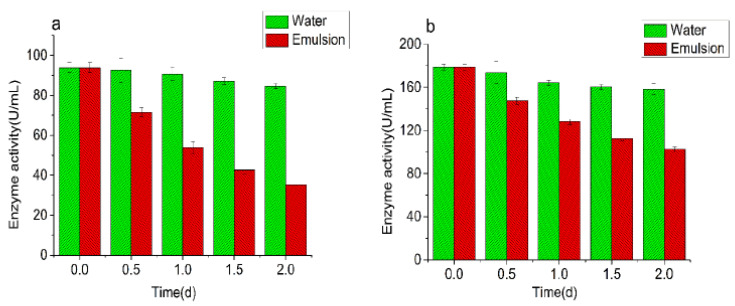

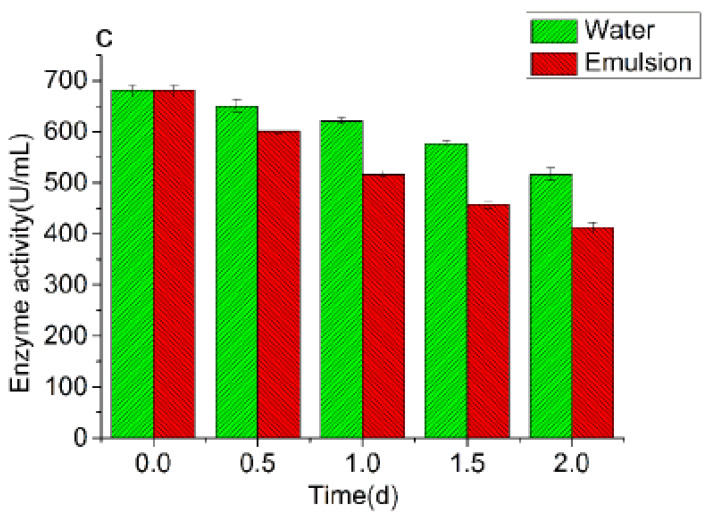
Enzyme activity changes in the water and emulsion containing different concentrations of CAT (**a**) 0.50 μmol/L, (**b**) 1.00 μmol/L, and (**c**) 5.00 μmol/L).Note: “d” in the figure is referred to day.

In order to explore the relationship between demulsification and CAT enzyme activity, two control experiments were conducted. One was to compare the effects of inactivated CAT and normal CAT on the turbidity of the emulsion (Figure 7), and the other was to compare the activity changes of CAT in emulsion and water (Figure 8). The initial turbidity values of the inactivated CAT group were 0.64–0.69. After 2 days incubation, the values were 0.61–0.62, indicating that the turbidity changed little in the emulsion treated with inactivated CAT. The initial turbidity values of the normal CAT group were 0.64–0.69, after 2 days of incubation, it decreased significantly, down to 0.12–0.14. The initial activity of the CAT in the water solution with 0.50 μmol/L, 1.00 μmol/L, and 5.00 μmol/L of CAT were 93.8 U/mL, 178.6 U/mL, and 680.6 U/mL, respectively. After 2 days of incubation, they were 84.6 U/mL, 158.2 U/mL, 516.8 U/mL, respectively, indicating that the CAT activity in water changed little. Yet, the CAT activity in the emulsion groups dropped significantly. After 2 days of incubation, it dropped to 35.2 U/mL, 102.5 U/mL, and 412.5 U/mL, respectively. These results indicated that the destructive effect of CAT on emulsion was not the interaction between protein and oil, but was closely related to the enzyme activity of CAT.

#### 3.3.2. CAT Activity and Lipid Oxidation

**Figure 9:**
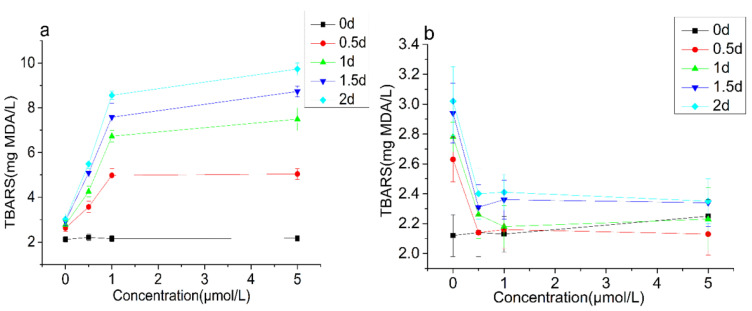
Thiobarbituric acid reactive substances (TBARS) changes of the emulsion treated with CAT (**a**) and BSA (**b**). Note: “d” in the figure is referred to day.

**Figure 10:**
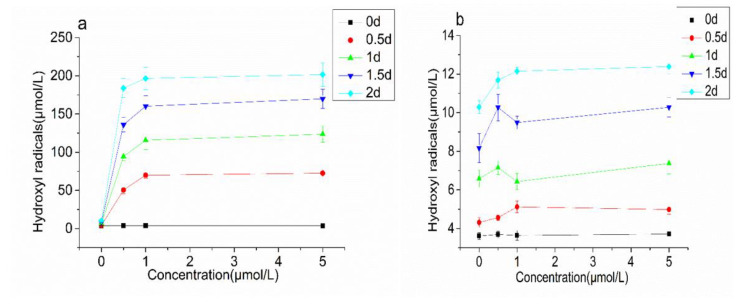
Hydroxyl radicals changes of the emulsion treated with CAT (**a**) and BSA (**b**).

In order to explore whether the demulsification effect of CAT was related to the lipid oxidation, TBARS and hydroxyl radicals in the emulsion were measured (Figure 9 and Figure 10). The TBARS value represents the content of the secondary oxidation product-malondialdehyde (MDA) in lipid oxidation, and it is an important indicator for measuring the level of lipid oxidation. The TBARS value increased rapid with the extension of the incubation time. When the concentration of CAT was below 1.00 μmol/L, the increasing speed of TBARS accelerated with the increase of CAT concentration. When the concentration of CAT was over 1.00 μmol/L, the increasing speed of TBARS correlated little with the concentration of CAT. Hydroxyl-free radicals are a kind of reactive oxygen free radicals with strong oxidizing ability. The accumulation of hydroxyl-free radicals can cause irreversible damage to cell membranes, DNA, and proteins, and affect the normal biological functions of the body. The initial content of hydroxyl radicals was 3.61–3.75 μmol/L. With the extension of the incubation time, the content increased rapidly. After 2 days of incubation, the content of hydroxyl radicals reached about 200 μmol/L. However, the changes of TBARS and hydroxyl radicals in the BSA group were not obvious.

## 4. Discussion

In this paper, a stable Antarctic krill oil emulsion was prepared using Tween-80 as the emulsifier, an oil concentration of 1%, and a ratio of surfactant to oil of 1:5. Subsequently, the effect of CAT on the characteristics of emulsion during storage was studied through visual observation, microscopic morphology observation, turbidity and stability, particle size, and ζ-potential, and these effects were compared with those of SOD and BSA. Demulsification and lamination were observed in the emulsion treated with CAT after 2 days of incubation while emulsion treated with SOD and BSA remained stable even after 7 days of incubation. Next, two experiments were designed to study the relationship between the CAT enzyme activity and its destructive effect. One compared the influence of inactivated CAT and normal CAT on turbidity changes of the emulsion and the other compared the enzyme activity changes of CAT in aqueous solution and the emulsion. The results indicated that the effect of CAT on the emulsion was not just the effect between protein and oil but the enzyme activity was also involved. Finally, the effect of CAT on the lipid oxidation in the emulsion was evaluated through the TBARS and hydroxyl-free radicals. The results showed that CAT could significantly promote the oxidation reaction in the emulsion. All these results indicated that CAT was not suitable for the removal of residual H_2_O_2_ in Antarctic krill oil emulsions. Moreover, it is helpful to avoid the contact of Antarctic krill oil emulsion and CAT during the processing of Antarctic krill emulsion.

CAT and SOD are both important enzymes in the human redox system [20]. The main physiological function of CAT is to catalyze the decomposition of hydrogen peroxide into water and oxygen, while SOD could catalyze the disproportionation of superoxide anion free radicals to generate oxygen and hydrogen peroxide. CAT and SOD play important roles in maintaining the balance of the redox system [21,22]. BSA is a protein used widely in biochemical experiments and it is a typical non-enzymatic protein [23]. The results of this investigation indicated that CAT could promote lipid oxidation of Antarctic krill oil emulsion and destroy the stability of the emulsion, while SOD and BSA had no significant effect. Comparing these three proteins, CAT is the only one with iron porphyrin, which is the center of CAT enzyme activity. The function of CAT is actually dependent on the heme-mediated electron transfer system. Iron porphyrin is likened to a “temporary storage tank of electrons”, which could complete the transfer of electrons according to the needs of the reaction [24,25]. When CAT functioned, the iron element continuously switched between low and high valence, which increased the risk of electron escape. Free electrons entered the emulsion and induced lipid oxidation, thereby destroying the stability of the emulsion [26,27]. However, in this experiment, hydrogen peroxide was not added to the emulsion, and no oxygen release was observed. Therefore, we ascribed this to the perhydroxyl radicals or H_2_O_2_-like molecules on the oil/water interface. In this study, we found that CAT could destroy the stability of Antarctic krill oil emulsion and began to elucidate the mechanism from the perspective of enzyme activity center and lipid oxidation. However, more research needs to be carried out to further prove this phenomenon and further elucidate the mechanism; for example, whether CAT has the same effect on other oil emulsion such as fish oil emulsion and olive oil emulsion. Whether other proteins containing iron porphyrin have the same effect on Antarctic krill oil also needed to be explored.

## 5. Conclusions

This article originated from the application of CAT in the removal of residual H_2_O_2_ in milk and juice after sterilization processing. However, the key to the enzymatic activity of CAT is the iron porphyrin group. Electron escape is prone to take place during the functioning of CAT. Free electrons can promote the oxidation and rancidity of oils. Based on this, we proposed that CAT might not be suitable for the removal of residual H_2_O_2_ in Antarctic krill oil emulsion. The results of this article also confirmed this, but more systematic studies need to done to further explore the mechanisms underlying this effect.

## Data Availability

Data are available upon request.

## Supplementary Materials

The following are available online at https://www.mdpi.com/article/10.3390/foods10112797/s1. Table S1: Fatty acid composition analysis of Antarctic krill oil (>1%); Table S2: The main functional ingredients of Antarctic krill oil; Table S3: Effects of different emulsifiers on the properties of the emulsion; Table S4: Effects of different oil concentrations on the properties of the emulsion; Table S5: Effects of different ratios of emulsifier to oil on the properties of the emulsion.
