# Supplementary material for: Preparation of Antarctic Krill Oil Emulsion and Its Stability under Catalase Treatment
Source: Foods. 2021 Nov 13;10(11):2797. doi: 10.3390/foods10112797 (PMC8625452; doi:10.3390/foods10112797)
Supplement: Supplementary file 1 [file foods-10-02797-s001.zip › foods-1395922-supplementary.pdf]

Table S1 Fatty acids composition analysis of Antarctic krill oil (>1 %)

Name	Content
C14:0	6.12±0.81 %
C16:0	21.01±1.34 %
C16:1(cis,n-7)	6.74±0.25 %
C17:1(cis,n-10)	1.10±0.05 %
C18:0	1.53±0.08 %
C18:1(cis,n-9)	11.31±0.65 %
C18:2(cis,n-6)	2.05±0.09 %
C18:3(cis,n-3)	2.97±0.06 %
C20:5(EPA)	22.78±0.55 %
C22:6(DHA)	13.71±0.26 %
∑SFA	28.66 % (estimated values)
∑MUFA	19.15 % (estimated values)
∑PUFA	41.51 % (estimated values)
EPA+DHA	36.49 % (estimated values)

Table S2 The main functional ingredients of Antarctic krill oil

Name	Content
Phospholipid	52.97±2.50 g/100g
Astaxanthin	8.21±0.81 mg/kg
Totaxin	4.25±0.50 mg/100g
Vitamin A	2.17±0.02 mg/100g
Cholesterol	7.19±0.55 mg/g

Table S3 Effects of different emulsifiers on the properties of the emulsion

	Turbidity	Stability (%)	particle size (nm)	ζ-potential (mV)
Tween-80	0.56±0.02	75.16±7.00	145.10±10.10	-40.60±3.00
Lecithin	0.45±0.05	42.37±4.00	378.60±15.30	-68.20±6.00
Span-20	0.64±0.03	69.19±6.00	184.20±13.00	-35.10±4.00

Table S4 Effects of different oil concentrations on the properties of the emulsion

	Turbidity	Stability (%)	particle size (nm)	ζ-potential (mV)
0.25%	0.16±0.01	56.75±5.00	1865.10±75.30	-10.20±0.50
0.50%	0.31±0.03	63.21±6.00	792.40±20.30	-20.60±0.90
1.00%	0.76±0.05	76.92±6.00	184.20±13.00	-26.10±1.20
2.50%	1.07±0.09	60.19±6.00	1025.30±30.60	-28.30±0.90
5.00%	2.13±0.10	41.76±4.00	2008.60±75.60	-30.20±2.10

Table S5 Effects of different ratio of emulsifier to oil on the properties of the emulsion

	Turbidity	Stability (%)	particle size (nm)	ζ-potential (mV)
1:10	0.83±0.05	36.79±5.00	253.20±9.60	-16.40±1.20

1:5	0.59±0.03	70.56±6.00	210.30±3.50	-33.50±1.50
1:1	0.75±0.05	68.34±5.00	146.50±4.00	-30.50±0.80
5:1	0.83±0.03	50.12±5.00	798.40±15.60	-25.30±0.60
10:1	1.05±0.04	32.18±3.00	1532.80±23.10	-22.60±0.90
